# Towards precision pain management in veterinary practice: opportunities and barriers

**DOI:** 10.3389/fvets.2025.1658765

**Published:** 2025-08-15

**Authors:** Jade-Lily C. Jonovski, Elouise K. Bacon, Brandon D. Velie

**Affiliations:** Equine Genetics and Genomics Group, School of Life and Environmental Science, University of Sydney, Sydney, NSW, Australia

**Keywords:** pharmacogenetics (PGx), analgesia, veterinary genomics, pharmacology, personalised medicine

## 1 Introduction

The capacity to tailor medical treatments to individual genetic profiles is steadily revolutionising modern healthcare. Known as precision medicine, this approach has been used to improve patient outcomes through a variety of medical applications ranging from drug choice to chronic disease management (1–13). Precision medicine seeks to end the “one size fits all” and “trial and error” approaches of medical treatments, minimising adverse effects and improving therapeutic efficacy to ultimately optimise patient outcomes (1–4, 14).

Serving as a cornerstone of precision medicine, pharmacogenetics facilitates the customisation of drug therapy based on an individual's genetic constitution for drug response (1–4, 14–16). For example, in humans, women with a polymorphism in the cytochrome P450 (*CYP*) gene family, specifically *CYP2D6*, are advised to pursue alternative treatments for early-stage hormone-receptor positive breast cancer rather than the gold-standard drug tamoxifen. This advice is given based on a loss of function mutation in *CYP2D6* resulting in deficient enzyme activity for tamoxifen metabolite catalysis and thus exhibiting poorer clinical outcomes and greater risk of breast cancer recurrence (17, 18). The applicability of these findings exemplifies how pharmacogenetic insights can directly inform clinical decision making and improve therapeutic outcomes; yet in veterinary species, pharmacogenetic approaches remain rare in routine practise.

Although variability in drug response at a species, breed, and individual level is well documented, much of veterinary pharmacogenetics remains in the early stages of research and validation (1, 15, 19). Current veterinary pharmacotherapy relies heavily on standardised dosing regimens based on population averages, disregarding individual variation in pharmacokinetic and pharmacodynamic parameters (13, 15, 16, 19). Breed-specific drug sensitivities, notably in herding breeds such as Collies, have spearheaded the early adoption of pharmacogenetic principles in veterinary practise, with further advances drawn heavily from human and rodent models predominantly in oncology (1, 5–12, 16, 20, 21). Consequently, research has sidelined broader therapeutic areas such as analgesia or chronic disease management.

Despite the overall lag in clinical adoption, several notable examples illustrate that pharmacogenetic principles are not merely theoretical in veterinary medicine. Mutations in genes such as ryanodine receptor 1 (porcine, canine, equine), ATP-binding cassette B-1 (*ABCB1*; canine), and various *CYP* (canine, equine) genes have been implicated in the occurrence of adverse effects and variability in drug clearance (1, 10, 22–51). Although clinically relevant, the limited scope identified in current pharmacogenes research highlights the vast opportunity to expand precision medicine principles into fields such as pain management, where failure to individualise therapy can have profound welfare implications.

## 2 Advancing veterinary pain management

Pain is a fundamental biological mechanism that serves both adaptive and maladaptive roles in animals. Adaptive pain functions as an essential protective response, alerting an individual to potential or actual tissue injury and stimulating behaviours that serve to minimise further harm (52–56). Conversely, maladaptive pain arises when the nociceptive system itself becomes dysfunctional (52–56). Pain manifesting without protective purpose profoundly reduces quality of life and presents significant challenges for veterinary practitioners (52–57). Since adaptive and maladaptive pain often coexist, the aim of veterinary pain management is not to eliminate all nociceptive input but rather to reduce maladaptive pain while preserving protective pain responses (52, 53, 56, 58).

Ensuring effective pain management is integral to improving patient welfare and reducing morbidity (57–61). Traditional approaches to pain management predominantly rely on pharmaceutical interventions. Nonsteroidal anti-inflammatory drugs, opioids, local anaesthetics, and alpha-2 adrenergic receptor agonists remain the foundations of analgesic protocols across species (53, 54, 57, 58). Selection of drugs is purpose-driven, guided by standard industry practise and clinician discretion, while dosage rates are shaped by population-wide efficacy and adjusted for body weight. Such approaches, while pragmatically necessary in the absence of individualised data, implicitly assume uniform pharmacodynamic responses among patients. Clinical observations and emerging pharmacogenetic research stand to increasingly challenge these practises (1–7, 9, 10).

Veterinary clinicians frequently encounter variability in analgesic efficacy, adverse drug reactions, and duration of action even among patients of the same species, breed, and size (21). Nevertheless, the prevailing model for analgesic selection and dosing remains largely reactive rather than predictive, with adjustments typically made only after the emergence of adverse effects or overall treatment failure (53, 54, 57, 58). Such reactive approaches risk prolonged animal suffering and delay effective intervention, undermining trust between owners and veterinary professionals (56–58). Precision medicine offers an opportunity to transform veterinary pain management from a reactive to a proactive discipline. Although gene-mediated analgesic response in animals remains minimally explored, genetic regions have been implicated in the delivery and metabolism of analgesic drugs.

*ABCB1* is one of few veterinary pharmacogenes associated with variability in analgesic drug response. P-glycoproteins are integral to drug transport across the blood-brain barrier, with mutations associated with increased central nervous system sensitivity to opioid analgesics in canines (1, 10, 30, 32, 33, 38, 39). Higher occurrence of the mutation within Collies and other herding breeds has facilitated earlier clinical exploration of precision approaches to analgesia (1, 30–39).

Similarly, the CYP gene family has been linked to drug metabolism across humans and veterinary species. Human *CYP2D6* is associated with over 100 alleles, resulting in four metabolic phenotypes for the O-demethylation of codeine into morphine, and further morphine into morphine-6-glucuronide, a metabolite with increased analgesic potency (10). In canines, an identified *CYP2D6* orthologue is associated with tramadol variability and is being explored in relation to deracoxib toxicity (45, 59, 60, 62). Further, in equines, two distinct metabolic phenotypes for codeine O-demethylation have been linked to the *CYP2D6* orthologue (47–51).

Despite early findings, a substantial knowledge gap persists. Deeper understanding of the underpinning genetic factors in analgesic response is needed to refine therapeutic strategies. To achieve truly effective and humane pain management, veterinary medicine must move beyond traditional standardised frameworks and embrace the development of precision-guided protocols. Nevertheless, without the genetic basis to guide decision making, precision analgesia is inhibited.

## 3 Barriers to clinical uptake

While precision-guided analgesia holds clear promise, its clinical implementation in veterinary medicine is met with substantial obstacles. Unlike human medicine, where pharmacogenetic integration has benefited from public health funding and pharmaceutical sector support, veterinary pharmacogenetics lacks comparable financial support (1, 5–11, 16, 61). Genetic testing remains prohibitively expensive for many animal owners, and in the absence of insurance coverage or decreased costs, widespread adoption is unlikely without incentivising action (1, 5–11, 16, 61).

Economic constraints extend beyond clinical settings, impacting research momentum. A lack of funding hampers large-scale studies needed to map genotype-phenotype relationships relevant to analgesic response at species- and breed-specific levels. As a result, pharmacogenes remain poorly defined across veterinary species, and many genes of interest lack comprehensive pharmacokinetic and pharmacodynamic data. Additionally, the current evidence is insufficient to convincingly demonstrate improved clinical outcomes (1, 5–11, 14, 16). Consequently, the development of standardised clinical guidelines remains a significant challenge. Without standardised protocols, clinicians may struggle to incorporate genetic insights effectively into therapeutic decisions, exacerbating the lack of research into clinical outcomes (1, 5–11, 16).

Moreover, pharmacogenetic phenotypes are often difficult to identify without pharmacokinetic or pharmacodynamic testing. In the absence of genetic data, clinicians are unable to identify affected individuals, as phenotypic indicators of genetic variants are often not observable. There is poor justification for precision approaches in time-sensitive cases when veterinarians are unable to readily obtain genotype data (1, 61).

Compounding this is the reality that many veterinarians rarely have access to genotype information. Unlike human medicine, where pharmacogenetic data may be embedded into electronic health records, veterinary clinicians often rely on owner curiosity and commercial genomics companies whose services lack regulatory oversight for validation and quality control standards (1, 5–11, 16, 63). Even when genetic data is available, improving genetic literacy among veterinarians must be a parallel priority. Without foundational and continuing education in how to interpret and apply pharmacogenetic data, even the best research and tools will struggle to gain meaningful clinical traction (63, 64). Efforts to centralise genetic information, such as the Online Mendelian Inheritance in Animals, offer academic value but are not currently structured for practical clinical use (16, 63, 65). Without high-quality testing and genetic resources readily available, precision-guided analgesia presently remains difficult to integrate in routine practise (1, 5–11, 16).

These systemic challenges underscore the need for broader infrastructure. Without such foundations, the potential of pharmacogenetics to advance precision medicine in veterinary care will not be fully realised. Only through systemic collaboration can the field move from isolated findings to routine, evidence-based precision pain management.

## 4 Towards practical integration

Despite these barriers, the future of precision-guided pain management in veterinary medicine is increasingly promising. Several emerging trends in pharmacogenetics point towards its increasingly feasible integration. Advances in genomics, bioinformatics, and clinical pharmacology are converging to create novel opportunities for more effective and individualised analgesic strategies across species.

One of the most exciting prospects for pharmacogenetics lies in the identification of genes that utilise visually apparent phenotypic markers as proxies for underlying genetic variation. Variants in the melanocortin 1 receptor (*MC1R*) gene, most famously associated with red hair and fair skin in humans, are known to be associated with altered levels of nociceptive regulation (66–68). Particularly in response to opioids, humans and rodents with an *MC1R* variant typically exhibit increased sensitivity (66–69). In equines, preliminary research has also begun exploring *MC1R* variants linked to the chestnut coat colour and variability in opioid metabolism (70). The existence of visually recognisable phenotypes tied to quantifiable variability in drug response offers a pragmatic entry point for the integration of pharmacogenetics into clinical practise. If validated, this approach could facilitate the prediction of analgesic needs by bypassing the immediate requirement for genetic testing, allowing more widespread clinical integration of precision pain management.

Where clinically relevant pharmacogenes have been identified, the next step is to ensure that genetic information is accessible at the point of care. The development of genetic arrays for pharmacogenes present in a significant proportion of veterinary species populations may allow for the testing of individuals at birth. This approach could integrate results and clinical interpretation into early health records, ensuring consistent access to genotype-informed guidance throughout the animal's life rather than adapting as sensitivities arise. Greater accessibility to interpretation and results will be able to aid practitioner confidence in pharmacogenetic applications (1–4, 16, 18, 64).

Ultimately, capitalising on these opportunities will require continued investment in species-specific pharmacogenetic research, validation of clinically actionable markers, and development of accessible, cost-effective testing platforms where phenotypes are not easily observable. With coordinated effort, precision-guided pain management could shift from a theoretical possibility to a standard of care, offering substantial improvements in therapeutic efficacy and patient welfare across veterinary medicine.

## 5 Discussion

Precision medicine offers a transformative opportunity to enhance the efficacy, safety, and individualisation of veterinary treatments. Pain management stands to particularly benefit from the integration of pharmacogenetics, offering a pathway to move beyond the conventional trial-and-error approach that currently defines analgesic therapy. Although early discoveries, such as *ABCB1* and *CYP2D6*, have illustrated the clinical relevance of pharmacogenetics in veterinary species, translation into routine practise remains slow.

Future progress will require interdisciplinary collaboration between clinicians, researchers, and geneticists to develop relevant genetic testing, establish evidence-based guidelines, and incorporate insights into everyday practise. Emerging opportunities, such as the identification of observable phenotypic markers, offer exciting prospects to bridge this gap. Investment in scalable tools, including pharmacogenetic arrays and clinical decision-support systems will be essential to operationalise these advances on a wide scale.

By addressing the current barriers and continuing to invest in foundational and translational research, veterinary precision medicine can shift from theory to practise. The ultimate vision is a veterinary healthcare model where treatments are tailored beyond species- and breed-specific generalisations. Such a transformation would not only optimise therapeutic outcomes but also substantially elevate the standard of care and welfare across all animal populations. Precision-guided veterinary pain management is no longer a distant goal but a realistic clinical objective which demands multidisciplinary collaboration to realise its full potential.
