# Correction: Towards precision pain management in veterinary practice: opportunities and barriers

**DOI:** 10.3389/fvets.2025.1687598

**Published:** 2025-09-05

**Authors:** Jade-Lily C. Jonovski, Elouise K. Bacon, Brandon D. Velie

**Affiliations:** Equine Genetics and Genomics Group, School of Life and Environmental Science, University of Sydney, Sydney, NSW, Australia

**Keywords:** pharmacogenetics (PGx), analgesia, veterinary genomics, pharmacology, personalised medicine

In the published article, there was an error in the title. Instead of “**Towards precision pain management in veterinary practise: opportunities and barriers**”, it should be “**Towards precision pain management in veterinary practice: opportunities and barriers**”.

In the published article, the name of one of the authors was incorrectly spelled in the reference for Jonovski J-LC, Bacon EK and Velie BD (2025) Towards precision pain management in veterinary practice: opportunities and barriers. *Front. Vet. Sci*. 12:1658765. doi: 10.3389/fvets.2025.1658765 as Jonovsk. It should be Jonovski.

The original article has been updated.

